# Higher neutrophil-lymphocyte ratio predicts poor response to repetetive transcranial magnetic stimulation(rTMS) in treatment resistant depression

**DOI:** 10.1007/s00406-025-01977-2

**Published:** 2025-03-05

**Authors:** Buket Koparal, Havva Nur Temizkan, Muhammed Hakan Aksu, Rukiye Filiz Karadağ

**Affiliations:** Gazi University Faculty of Medicine, Gazi Universitesi Tip Fakultesi, Ankara, Türkiye

## Abstract

Repetitive transcranial magnetic stimulation (rTMS) has emerged as a noninvasive therapy for treatment resistant depression (TRD). The results of studies on the mechanism of rTMS and the predictive parameters for determining which patients will respond to rTMS are inconclusive. This study aims to investigate the relationship between pre-treatment systemic inflammatory markers and the response to rTMS in TRD patients. We retrospectively reviewed 86 patients with TRD who received 10 Hz rTMS to the left dorsolateral prefrontal cortex (DLPFC) at Gazi University between June 2017 and June 2023. Inflammatory markers, including neutrophil-lymphocyte ratio (NLR), platelet-lymphocyte ratio (PLR), and systemic immune-inflammatory index (SII), were evaluated. Treatment response was assessed using the Montgomery-Åsberg Depression Rating Scale (MADRS), with a ≥50% reduction in MADRS score and a score ≤8 at the end of treatment considered as a positive response. Patients divided in to two groups accoording to rTMS response. 53 patients were responders and 33 patients were non-responders. Significant differences in inflammatory parameters were observed, with non-responders showing higher NLR (*p*=0.001), PLR (*p*=0.008), and SII (*p*=0.002) values. Logistic regression analysis revealed that higher NLR was significantly associated with a poorer response to rTMS (OR=0.373, *p*=0.022). Additionally, early improvement in MADRS score in the first week predicted overall treatment outcome (OR=1.070, *p*<0.001). Our findings suggest that systemic inflammation plays a role in TRD and that higher pre-treatment NLR is associated with a poorer response to rTMS. Largerscale studies are needed to further understand the mechanisms and improve treatment strategies for TRD patients.

## Introduction

Major depressive disorder (MDD) is an important medical condition that causes significant impairment in interpersonal, social and occupational functioning. Depression can be caused by a multitude of factors, including neurotransmitter dysfunction, nervous system inflammation, irregularities in the body’s immune response, biological rhythms, cellular energy production dysfunction, excessive stress response activity (hypothalamic-pituitary-adrenal axis), oxidative stress, and environmental stressors [1, 2]. According to the 2017 report of World Health Organisation (WHO), it is estimated that aproximately 280 million people suffers from depression [3]. Despite advancements in pharmacological and psychotherapeutic interventions, an estimated 30% of diagnosed individuals do not respond adequately to standard treatments, known as treatment-resistant depression (TRD) [4].

Repetitive transcranial magnetic stimulation (rTMS) is a non-invasive treatment that modulates the brain’s activity via electromagnetic induction. rTMS employs a brief yet intense magnetic field to regulate the activity of specific areas within the cerebral cortex [5]. The efficacy of low-frequency (≤ 1 Hz) stimulation targeting the right prefrontal cortex or high-frequency (≥ 5 Hz) stimulation targeting the left prefrontal cortex in the treatment of depressive disorders, along with their clinical tolerability, was established in the early 2000s [6]. While TMS has demonstrated efficacy in alleviating depressive symptoms in a subset of TRD patients, response rates remain variable, and predictors of treatment outcomes are not yet fully understood [7, 8].

Inflammatory dysregulation is a significant factor in the pathogenesis of major depression, as demonstrated by both preclinical and clinical researches [9, 10]. Numerous studies have indicated that elevated levels of inflammatory markers are linked to the severity of depression and resistance to treatment [11]. Meta-analyzes on the connection between inflammatory mediators in depression often focus on proinflammatory cytokines such as IL-1, IL-6, TNF-α, C-reactive protein (CRP) and anti-inflammatory cytokine IL-10 [12–15]. Recently, there has been increasing interest in the exploration of biomarkers that have the potential to predict the therapeutic response tor TMS in TRD. Some studies found that pre-treatment levels of inflammatory markers or immune activation may predict the response to rTMS [16, 17].

The quantification of neutrophils, platelets, and lymphocytes in peripheral blood, along with their respective ratios such as neutrophil/lymphocyte ratio(NLR), platelet/lymhocyte ratio (PLR), systemic immun inflammatory index (SII) represents cost-effective and straightforward methods for assessing systemic inflammation [18]. NLR, reflecting the neutrophil-to-lymphocyte ratio, and PLR, indicating the platelet to lymphocyte ratio, are recognized markers of innate immune response and are widely acknowledged as indicative of overall systemic inflammation [19]. A recently introduced a new prognostic ndex, the SII, combines platelet, neutrophil, and lymphocyte counts, calculated as SII = Plateletx Neutrophil/Lymphocyte [20]. These ratios have demonstrated prognostic significance in various systemic conditions such as cardiovascular disease, sepsis, and cancer [21–24]. Recent investigations have extended the application of blood cell ratios to psychiatric disorders, revealing a positive correlation between NLR and depression severity [25]. There exists a dearth of research examining the NLR, PLR, or SII in the context of antidepressant treatment outcomes. The findings from existing studies about are currently inconclusive, presenting a heterogeneous landscape. Some studies have identified a positive correlation between higher NLR levels and improved clinical response to pharmacotherapy in patients with psychotic depression [26, 27]. Conversely, other studies indicate no significant alterations in these ratios among treated patients with bipolar disorder or those with various psychiatric diagnoses following electroconvulsive therapy (ECT).

To the best of our knowledge, no other study has investigated the relationship between rTMS treatment response and blood parameters which reflects inflammation from in TRD. In this study, we aimed to examine the relationship between peripheral inflammatory blood parameters and treatment response to rTMS in treatment resistant depression.

## Material and methods

### Procedure

This study was conducted using a retrospective review of 272 patients who met the criteria for major depression and received standard clinical treatment of 10 Hz rTMS to the left dorsolateral prefrontal cortex (DLPFC) at Gazi University Faculty of Medicine Department of Psychiatry, between June 2017 and June 2023.

The Neurosoft brand Neuro-MS/D magnetic stimulator device is utilized for rTMS application. Our treatment protocol involves applying frequency of 10 Hz, power(intensity) %120 of the motor threshold, 3000 pulses per session and five sessions per week. The stimulation targets to the left dorsolateral prefrontal cortex (DLPFC).

A data form consisting of socio-demographic, clinical, and laboratory characteristics was formed and recorded by the researchers. Ethical approval was obtained from the Gazi University Ethics Committee (Research code: 2023 − 900).

### Sample

Patients who are unresponsive to at least 2 antidepressants at adequate doses for minimum four weeks are defined TRD. Patients are evaluated by clinicians before treatment, treatment response and side effects are assessed using baseline and weekly by scales. During treatment, patients continue their current pharmacological treatments, with no medication changes made unless absolutely necessary.

Patients aged 18 and above, who underwent a complete blood count before the 14-day period preceding rTMS treatment, received a minimum of 20 sessions of rTMS over a four-week, and had complete pre- and post-treatment symptom severity scales (Montgomery-Asberg Depression Rating Scale) were included to study. A total of 128 patients who had bipolar depressive disorder or/and co-existing psychiatric disorder (except for smoking addiction), 5 patients who were found to have lymphopenia, and 4 patients who had an active infection during rTMS treatment were not included to the study. 49 patients who had not completed at least 20 sessions of rTMS treatment were also excluded from the study. Patients with diabetes, chronic smoking, hypertension, and known inflammatory diseases were not excluded from the study and recorded to data form.

### Clinical assesments

The Montgomery-Åsberg Depression Rating Scale (MADRS): It was developed to assess the severity of depressive symptoms and measure changes resulting from treatment. Scores between 9 and 29 indicate mild depression, scores between 30 and 36 indicate moderate depression, and scores above 36 indicate severe depression. A Turkish validity and reliability study has been conducted [28].

MADRS score change was reported as a percentage change from baseline. Response criteria were ≥ 50% reduction in Montgomery-Asberg Depression Rating Scale (MADR-S) score and a score ≤ 8 at end-of-treatment.

### Peripheral inflammatory markers

Neutrophile-lyphocyte ratio (NLR) was calculated as the ratio of neutrophil to lymphocyte counts, platelet-lymhocyte ratio (PLR) was calculated as the ratio of platelet to lymphocyte counts. The SII was calculated as follows: (platelet count × neutrophil count)/lymphocyte count.

### Statistical analysis

Descriptive statistical methods were utilized to present a comprehensive overview of both continuous and categorical data. The data was displayed in the form of mean and median values, standard deviations, minimum and maximum values, frequencies, and percentages. The aim of this data presentation was to offer a detailed description of the demographic and clinical profiles, as well as the laboratory values of the patients included in our study. The comparative analysis between treatment respondent and non-responder patients involved Chi-square tests and the Mann-Whitney U test for categorical and parametric variables, respectively. To explore potential relationships between continuous variables, Spearman correlation analyses were conducted. Furthermore, a multivariable logistic regression employing the likelihood ratio (LR) method was utilized to examine the predictors of response to rTMS. Variables demonstrating a significant relationship (*p* < 0.05) in the univariate analyses were entered into the logistic regression analysis as independent variables. Regression analyses were adjusted to control for the effects of age, sex, smoking status, diabetes, hypertension, and known inflammatory diseases.

The statistical analyses were performed using IBM SPSS Statistics, version 26.0. *p* < 0.05 was considered at statistically significant.

## Results

As a result of the retrospective review, data from 86 patients were collected. The average age of all patients was 44.05 ± 14.713 years. The number of female patients was 60 (69.80%). The sociodemographic and clinical characteristics of the patients are presented in Table 1.

**Table 1:** Sociodemographic and Clinical Characteristics and Comparison of Sociodemographic and clinical characteristics between responder and non-responder patients

		Responders (*n* = 53)	Nonresponders (*n* = 33)		
	Mean ± SD	Median (min-max)	Median (min-max)	u	p
Age, years	44,05 ± 14,713	42(19–72)	44(22–78)	793	0.469
Neutrophil-Lymphocyte ratio	2,05 ± 0,87	1.69 (0.95–3.56)	2.31 (0.98-6,12)	503.5	0.001
Platelet-Lymphocyte Ratio	124,17 ± 46,42	105.50 (11.50–243,85)	136.80 (50.71–280.00)	578	0.008
Systemic İmmune-İnflammation İndex (Log10)	5,70 ± 0,22	5.63 (4.89-6.00)	5.78 (5.32–6.28)	519	0.002
Duration of current depressive episode (month)	6,35 ± 7,88	3 (3–48)	3 (3–24)	856	0.857
Number of depressive episode	4,62 ± 3,86	3 (1–15)	3 (1–20)	773	0.359
MADRS scores before rTMS	31,94 ± 10,25	32.00 (13–54)	30.00 (16–53)	955	0.465
MADRS scores after rTMS	15,50 ± 11,91	9.00 (1–22)	21.00 (10–52)	108.5	< 0.001
First week MADRS score change(%)	32,87 ± 23,57				
End of rTMS treatment MADRS score change(%)	52,08 ± 30,49				
	n(%)	n(%)	n(%)	X^2^	p
Gender, female	60(69,80)	25(75,80)	35(66,00)	0,911	0,340^a^
Marital Status, Married	40(48,80)	16(48,50)	24(49,00)	0,002	0,965^a^
Previous history of rTMS	10(11,60)	1(3,00)	9(17,00)	3,852	0,081^b^
Inpatient history	37(43,00)	13(39,40)	24(45,30)	0,288	0,592^a^
Previous history of ECT	12(14,00)	6(18,20)	6(11,30)	0,160	0,524^b^
Suicide attempt history	30(34,90)	10(30,30)	20(37,70)	0,495	0,482^a^
Psychiatric illness history (1st-degree relatives)	32(37,20)	12(36,40)	20(37,70)	0,160	0,898^a^
Tobacco Use	39(45,30)	13(39,40)	26(49,10)	0,766	0,381^a^
Diabetes mellitus and/or hypertension	29(33,70)	12(36,40)	17(32,10)	0,167	0,683^a^
Inflammatory diseases	10(11,60)	2(6,10)	8(15,10)	1,615	0,305^b^
Medications					
Antideppressant alone	19 (22,2)				
Combination of antidepressants	14 (16.2)				
Combination of antidepressants and mood stabilizators/antipsychotics	53 (61.6)				

U, Mann-Whitney U Test; n, number; SD, standard deviation

^a^Chi-square ^b^ Fisher exact

Patients were divided into two groups based on their responses to rTMS treatment: responders and non-responders. Groups were compared in terms of sociodemographic and clinical characteristics in Table 1. No significant differences were found between the groups in terms of age (U = 793, *p* = 0.469), gender (χ²=0.911, *p* = 0.340), and marital status (χ²=0.002, *p* = 0.965). Significant group differences were present among the inflammatory parameters. The NLR (U = 503.5, *p* = 0.001), PLR (U = 578, *p* = 0.008), and SII (Log10) (U = 519, *p* = 0.002) values were statistically significantly higher in non-responders. There were no statistically significant differences between the groups regarding the duration of the current depressive episode (U = 856, *p* = 0.857), number of depressive episodes (U = 773, *p* = 0.359), previous history of TMS response (χ²=3.852, *p* = 0.081), inpatient history (χ²=0.228, *p* = 0.592), previous history of ECT (χ²=0.160, *p* = 0.524), suicide attempt history (χ²=0.495, *p* = 0.482), psychiatric illness history in first-degree relatives (χ²=0.160, *p* = 0.898), tobacco use (χ²=0.766, *p* = 0.381), diabetes mellitus and/or hypertension (χ²=0.167, *p* = 0.683), and inflammatory diseases (χ²=1.615, *p* = 0.305). There was no difference between the groups in terms of MADRS scores before TMS (U = 955, *p* = 0.465). As expected, there was a statistically significant difference between the groups in terms of MADRS scores after TMS (U = 108.5, *p* < 0.001).

The relationships between age, duration of the current depressive episode, number of depressive episodes, NLR, PLR, SII, and persentage change in MADRS score at the end of first week and at the end of rTMS treatment are presented in Table 2. Statistically significant correlation was found between at the end of treatment MADRS score change and NLR (*r*=-0.253, *p* = 0.019), PLR (*r*=-0.238, *p* = 0.027), SII (*r*=-0.247, *p* = 0.022), and First-week MADRS score change (*r* = 0.629, *p* < 0.001).

**Table 2:** Correlation analysis of continuous variables

		1	2	3	4	5	6	7	8
1.Age	r	1							
	p	.							
2.Duration of current depressive episode	r	0,133	1						
	p	0,223	.						
3.Number of depressive episode	r	0,067	-0,096	1					
	p	0,538	0,377	.					
4.Neutrophil-Lymphocyte ratio	r	0,076	0,039	-0,052	1				
	p	0,484	0,722	0,636	.				
5.Platelet-Lymphocyte Ratio	r	-0,042	0,087	-0,081	,549	1			
	p	0,702	0,426	0,460	< 0,001^*^	.			
6.Systemic İmmune-İnflammation Index (Log10)	r	0,061	0,026	-0,022	,815	,757	1		
	p	0,578	0,814	0,838	< 0,001^*^	< 0,001^*^	.		
7.First week MADRS score change	r	-0,029	0,066	-0,068	-,321	-0,159	-0,222	1	
	p	0,794	0,546	0,531	0,003^*^	0,143	0,040^*^	.	
8.End of rTMS treatment MADRS score change	r	-0,109	0,068	-0,100	-,253	-,238	-,247	,629	1
	p	0,318	0,540	0,361	0,019^*^	0,027^*^	0,022^*^	< 0,001^*^	.

*p < 0.05

Table 3 presents the predictors of response to repetitive transcranial magnetic stimulation (rTMS) based on a multivariable logistic regression analysis. NLR, PLR, SII, and first-week MADRS score changes (as a persentage value) are independent variables. The analysis includes 86 participants and identifies significant predictors such as the NLR and the change in the Montgomery-Åsberg Depression Rating Scale (MADRS) score at the first week of treatment.

**Table 3:** Predictors of response to rTMS: results of Multivariable Logistic Regression Analysis (*N* = 86)

	B	S.E.	Wald	df	Sig.	Exp(B)	95% C.I.for EXP(B)	
Neutrophil-Lymphocyte ratio	-0,986	0,429	5,284	1	0,022	0,373	0,161	0,865
First week MADRS score change	0,068	0,016	18,923	1	< 0.001	1,07	1,038	1,103
Constant	0,565	1,002	0,318	1	0,573	1,76		
Model Summary								
χ2 [df] (p-value)						42.612[2](*p* < 0.001)		
Hosmer and Lemeshow χ2 [df] (p-value)						9.308[8](*p* = 0.317)		
-2 Log likelihood						71.915		
Nagelkerke R2						0.531		
Overall percentage of correct predictions (%)						80.2		

The odds ratio (Exp(B)) for NLR was found to be 0.373 (95% CI: 0.161–0.865, *p* = 0.022). This indicates that an increase in NLR is associated with a decreased likelihood of responding to rTMS. Specifically, for each unit increase in NLR, the odds of a positive response to rTMS decrease by approximately 62.7% (1–0.373), assuming all other variables are held constant.

The odds ratio for the change in MADRS score at the first week was 1.070 (95% CI: 1.038–1.103, *p* < 0.001). This suggests that an improvement in the MADRS score in the first week increases the likelihood of a positive response to rTMS. Specifically, for each unit improvement in the MADRS score, the odds of a positive response to rTMS increase by 7%, assuming all other variables are held constant.

**Fig. 1:**
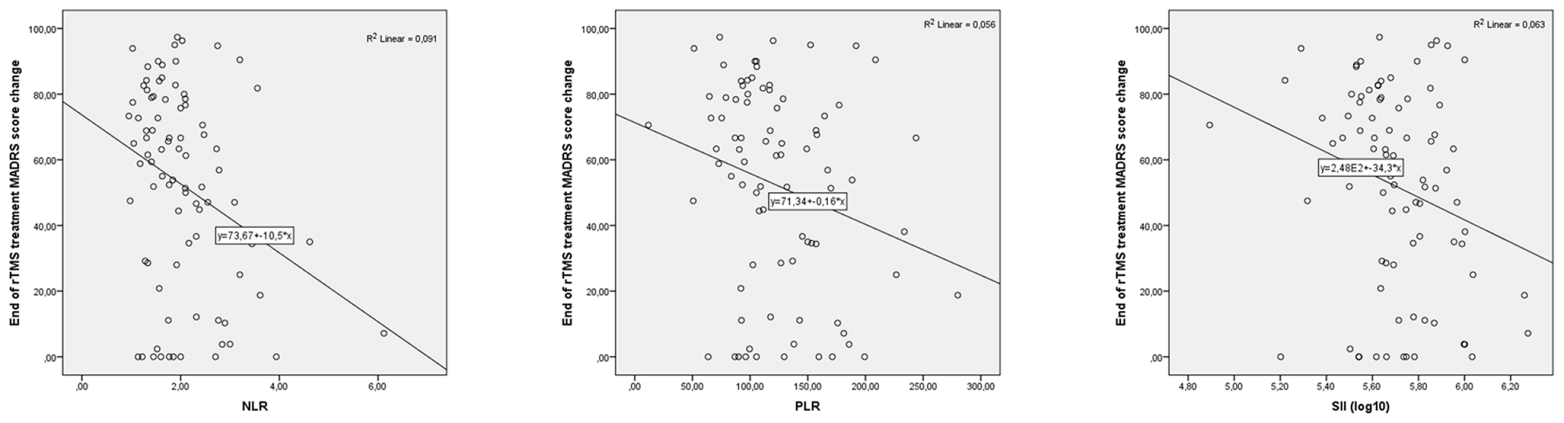
Correlations of blood parameters with end of rTMS treatment MADRS change

**Fig. 2:**
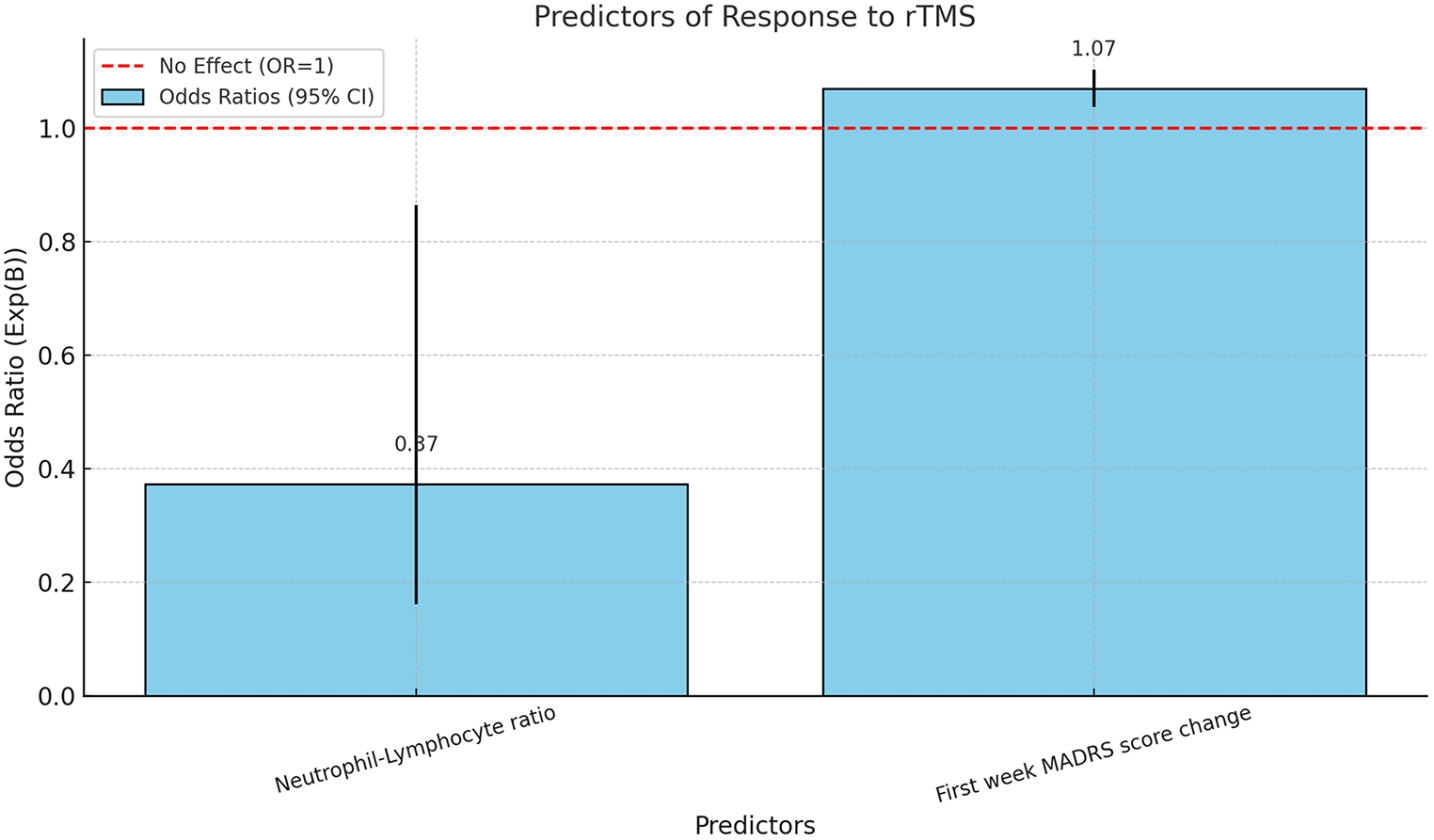
Predictors of rTMS response

The model demonstrates a good fit, as indicated by the χ² and Hosmer and Lemeshow test results, and explains approximately 53.1% of the variance in response to rTMS, with an overall correct prediction rate of 80.2%1(Figs 1, 2).

## Discussion

In this study, which aims to evaluate the response to rTMS treatment in relation to peripheral inflammatory parameters and other factors that may predict rTMS response in treatment resistant depression, our primary findings are consistent with the notion that the NLR and first week response to treatment plays a prognostic role in predicting rTMS response.

rTMS is an effective neuromodulation technique used in the treatment of TRD. Increasing evidence suggests that the efficacy of rTMS on depression is mediated through anti-inflammatory processes [29, 30]. By delivering magnetic pulses to specific brain regions, rTMS modulates neuronal activity, which may contribute to the reduction of inflammatory responses and the activation of anti-inflammatory pathways [5]. Although some hypotheses have been proposed regarding the mechanism of action, predicting which patients will respond to rTMS remains challenging. Studies on this subject have yielded mixed results; nonetheless, female gender, younger age, milder severity of depression, and shorter duration of depressive episodes have been associated with better treatment responses [31, 32]. In our study, no significant differences were found between responders and non-responders to rTMS treatment in terms of socio-demographic and clinical variables such as age, gender, number of depressive episodes, number of hospitalizations, and duration of depression. However, it was observed that the non-responder group had higher NLR, PLR, and SII values in univariate analyses. We also found that the response to rTMs treatment measured by the MADRS at the end of the first week is predictive of the overall treatment outcome consistent with the literature [33–35].

Most studies investigating the relationship between pre-treatment systemic inflammatory markers and treatment response have mainly focused on pharmacological interventions [26, 27]. In a study, higher baseline levels of the pro-inflammatory cytokines IFN-γ and CCL-2 were associated with poorer response to escitalopram monotherapy and adjunctive aripiprazol in major depressive disorders [36]. In another study, patients with higher pre-treatment NLR was not associated with symptom remission or the resolution of psychotic features in depressed patients [27]. The important point in these studies is that the patients included in the study were those with non-treatment-resistant major depression. According to these studies and the findings of our study, it is possible that systemic inflammation, as indicated by higher NLR, may be linked to poor response to both pharmacotherapy and rTMS treatment in patients with depression, regardless of treatment resistance. Inflammation may interfere with the efficacy of these treatments, possibly by altering neural plasticity and neurotransmitter function, both of which are critical for the response to pharmacological and rTMS interventions [37]. Our findings supports the hypotesis that systemic inflammation plays a role in treatment resistant depression [38].To the best of our knowledge, this study is the first to investigate the relationship between pre-treatment blood parameters which shows peripheral inflammatory markers and response to rTMS in patients with TRD.

In our study, a significant association was observed between pre-treatment neutrophil-to-lymphocyte ratio NLR and rTMS response, whereas no such relationship was identified for SII or PLR. Several factors may account for this finding. First, NLR combines two cell types—neutrophils and lymphocytes—that are directly involved in inflammatory processes, potentially providing a more accurate and sensitive reflection of systemic inflammation dynamics [39]. In contrast, the inclusion of platelets in PLR and SII, while valuable, introduces additional variability as platelets are involved in numerous physiological and pathological processes beyond inflammation, such as coagulation and vascular responses, making these markers less specific to inflammatory states [40]. Moreover, platelet function may exhibit considerable variability in conditions such as smoking, diabetes, and obesity, which could reduce the statistical significance of platelet-associated markers [41]. These factors might be contributed to the observed differences in the predictive value of these inflammatory parameters in our study.

There are limited studies investigating the association between inflammation and the response to neuromodulation therapies, such as electro-convulsive therapy (ECT) and TMS [16, 17, 42]. In a scoping review evaluating the relationship between rTMS treatment and immune-inflammatory markers in depression, a total of 12 studies were identified [5]. Among these, 7 studies investigated the relationship between rTMS treatment and immune markers in patients with treatment-resistant depression [17, 43–48]. Yılmaz et al. found an association between high pre-treatment CRP and IL-6 levels and non-response to rTMS treatment [17]. Valiuline et al. found that lower initial TNF-α levels are predictive for treatment response [43]. On the other hand, Stirton et al., in a study involving 48 patients, found associations between pre-treatment total and fragmented oxidized phosphatidylcholine (OxPC) levels and treatment response [16]. The researchers found that higher pre-treatment OxPC levels were associated with a better treatment response. Song et al. also reported that higher baseline antithrombin III (ATIII) levels predicted better rTMS treatment response [49]. Our findings support studies reporting that higher levels of inflammation are associated with a poorer response to rTMS treatment (*p* < 0.005). However, in some studies investigating different inflammatory markers, no relationship was observed between baseline inflammatory markers and rTMS response [43, 45, 46]. Discrepancies in different results across studies may be attributed to variations in the use of different biomarkers, sample sizes, and the inclusion or exclusion of other factors affecting inflammatory markers. For example, in five studies which investigate the relationship between rTMS response and immune inflammatory markers, patients were not treatment resistant. In our study, patients underwent pre-treatment evaluations, and rTMS was administered to those who had not achieved a sufficient response despite receiving at least two antidepressant treatments at adequate doses and lenghts [50]. The inclusion of very specific patient groups in some studies may also be a reason for different outcomes. For example, in the study conducted by Zhao, only treatment-resistant elderly patients were included [44]. In our study, we investigated conditions that could affect inflammatory markers, such as chronic smoking, inflammatory diseases, diabetes mellitus and hypertension; therefore, we did not find any differences between groups. This could be one reason for the differing results observed in studies examining the relationship between inflammatory markers and rTMS response. Studies involving patients with TRD had relatively small sample sizes, with a median of 25 patients and a range of 10 to 58 [5]. A relatively large sample size (*n* = 86) and the inclusion of only treatment-resistant depressive patients may explain the differences in results between previous research. However, these might also be interpreted as the strength of our study’s findings.

Although some studies have identified a relationship between rTMS treatment response and inflammatory markers, it is unlikely that the effects of rTMS are only mediated through immune or inflammatory mechanisms. A complex interactions including inflammation, oxidative stress, neurogenesis and neurodegeneration may be responsible for the therapeutic effect of rTMS. Nonetheless, our study’s findings have significance in suggesting that easily accessible and cost-effective methods such as measuring SII, NLR, and PLR, which are considered inflammation markers, along with closely monitoring treatment response early on, can help predict the outcome for TRD patients undergoing rTMS treatment. Our results regarding the predictive effect of both the first week’s treatment response on the treatment outcome and the predictive effect of higher levels of blood cell-derived inflammatory parameters on treatment response should be confirmed with larger sample-sized prospective studies. Thus, given the duration and cost of rTMS treatment, alternative treatments for example ECT might be considered earlier for the patients who show low response tor TMS in first week and have higher inflammatory markers.

The study has some limitations. Retrospective design is the main limitation of the study. Unfortunately, our study also lacked measures of post-treatment inflammatory markers and a healthy control group. These limitations make it challenging to establish the effect of rTMS treatment on inflammatory markers and may reduce the generalizability of our results. Although many conditions that could lead to low-level inflammation were noted, the lack of body mass index (BMI) data in patients means that obesity, a factor influencing inflammation, was not assessed, which is another limitations of our study. Well-designed large-scale prospective studies that consider all these limitations could provide valuable insights into the mechanism of action of rTMS.

In conclusion, the relationship between pre-treatment ƒinflammatory markers and treatment response rates in various psychiatric and physical illnesses has been the subject of numerous studies. This study suggests that monitoring inflammatory markers such as NLR before rTMS treatment may help identify patients who are less likely to respond, guiding treatment decisions. Investigating biological factors, particularly those affecting rTMS response, in larger sample studies will help us better understand the mechanism of action of rTMS.
